# An environmental analysis of public UAP sightings and sky view potential

**DOI:** 10.1038/s41598-023-49527-x

**Published:** 2023-12-14

**Authors:** R. M. Medina, S. C. Brewer, S. M. Kirkpatrick

**Affiliations:** 1 Department of Geography, University of Utah, Salt Lake City, UT 84112 USA; 2 United States Department of Defense, Washington D.C., 20301 USA

**Keywords:** Psychology and behaviour, Computational science, Natural hazards

## Abstract

Sightings of unidentified flying objects (UFOs) or unidentified anomalous phenomena (UAP) have been reported throughout history. Given the potential security and safety risks they pose, as well as scientific curiosity, there is increasing interest in understanding what these sighting reports represent. We approach this problem as an important one of the human experience and that can be examined through a geographical lens: what local factors may increase or decrease the number of sighting reports? Using a Bayesian regression method, we test hypotheses based on variables representing sky view potential (light pollution, tree canopy, and cloud cover) and the potential for objects to be present in the sky (aircraft and military installations). The dependent variable includes over 98,000 publicly reported UAP sightings in the conterminous United States during the 20-year period from 2001 to 2020. The model results find credible correlations between variables that suggest people see more “phenomena” when they have more opportunity to. This analysis is one of few investigations of UAP sighting reports at a national scale providing context to help examine individual reports. Given that these objects are labeled unidentifiable in the personal sense, there are many natural and/or human based explanations worth exploring.

## Introduction

There has been growing interest by the United States government in Unidentified Aerial Phenomena (UAP). Given the new focus on this potential security threat and the operational safety risks posed by these objects, the UAP Task Force was initiated on August 4, 2020 ^1^. This task force had a limited scope, authority, and resources to address the issue and was temporary in its duration. The Deputy Secretary of Defense gave direction to transition the UAP task force into the Airborne Object Identification and Management Synchronization Group (AOIMSG) on November 23, 2021 ^2^. Congressional legislation, however, overtook that direction and today’s All-Domain Anomaly Resolution Office (AARO) was established on July 20, 2022, as the single authoritative UAP Office with the DoD and tasked with leading and synchronizing a whole of government approach to the issue ^3^. The mission of the AARO is to: “synchronize efforts across the Department of Defense, and with other U.S. federal departments and agencies, to detect, identify and attribute objects of interest in, on or near military installations, operating areas, training areas, special use airspace and other areas of interest, and, as necessary, to mitigate any associated threats to safety of operations and national security. This includes anomalous, unidentified space, airborne, submerged and transmedium objects” ^3^. Supporting these efforts, this research team explores spatial patterns of publicly reported UAP sightings (analogous to UFO sighting reports in this research) from an open-source online dataset.

In the public 2021 Director of National Intelligence (DNI) report, research on UAP sighting reports between 2004 and 2021 leaves most of its 144 government-based reports unexplained, due to limited data. Only one sighting report was explained with high confidence and was found to be a deflating balloon ^4^. The follow-up 2022 DNI report indicates the number of governmental sourced reports rose to 510, with nearly half still unexplained. The DNI states that there is no single explanation for these UAP, with potential sources including clutter, commercial drones, national security threats, and other unexplained phenomena. Other early incarnations of government-based UFO research efforts (e.g., Project Sign in 1948, Project Grudge, then the most popular, Project Blue Book led by Dr. Allen Hynek in the 1950-1960s ^5^, and the following Condon Report funded by the U.S. Air Force and conducted at the University of Colorado) ended with about 5% of unidentified sightings ^6^. UAP research is often inconclusive, and our ability to explain these events seems to have become less easily resolved as our sensor technology has advanced and our air activity has increased.

Here, we ask three foundational research questions: (1) What is the viability of publicly offered data on UAP sighting reports? (2) Are there credible spatial patterns to these reports? and (3) If so, can these patterns be explained by physical and/or built environment factors? To answer these questions, we use UFO sighting report data from the National UFO Research Center ^7^. We model the total count of these reports over a 20-year period from 2001 to 2020, using environmental explanatory variables—light pollution, cloud cover, tree canopy cover, airports, and military installations. This model is intended to represent both the available view of the sky as well as the potential for airborne objects. We hypothesize that (a) factors limiting visibility will be negatively correlated with sighting reports, and (b) factors related to air traffic will be positively correlated, or simply that people will report sightings of UAPs where they have the most opportunity to see them. To our knowledge, this is the first attempt to understand how spatial variation in reports is linked to environmental variables. This analysis represents one of few attempts to examine this phenomenon at the national level and offers a starting point for a similar approach to be applied to U.S. Government data on UAP activity to help identify possible sources.

## History of UAP sighting research and Environmental Explanations

There has been little traditional academic research on UAPs. This is expected as there are always efforts to discredit scientific endeavors toward understanding this phenomenon ^8^, however we should not ignore the fact that many people throughout the world report having seen unknown and unexplainable objects in the sky. What research does exist, tends to rely on firsthand accounts, psychological explanations, or specific events, which limits the systematic analysis of large area patterns ^9–12^. Furthermore, verifiable data sources and questionable accounts have limited the rigor of previous work. Data availability for larger studies has been a longstanding issue. Recently in the U.S. there has been increased attention given to sightings by members of the military, or other government personnel. Databases of these events are now being kept by the AARO and the supporting Services, but these efforts only began in 2019, though they do hold information going back to 1996 ^13^. Congress has directed AARO to extend this research back to 1945.

An explanation for some UAP sightings is natural phenomena. For example, the planet Venus is often mistaken for a UAP. At times, it is seen close to the horizon and can shine through the trees to produce an irregular pattern of light and reflection ^14^. The second most likely explanation is human-made aircraft, ^15^ including various objects, such as weather balloons, originally believed to be responsible for the Roswell, New Mexico Case in 1947, arguably the most popular UAP case in U.S. popular culture. Follow up disclosures by the Air Force describe the activity responsible for the event as being a classified, multi-balloon project intended to detect Soviet nuclear tests ^16^. Current factors contributing to UAP sighting reports include the exponential growth in satellite and spacecraft launches and orbiters (e.g., SpaceX Starlink), as well as increased drone activity. The use of these and other modern technologies has likely led to increased UAP reports. The 2021 U.S. Office of the Director of National Intelligence’s Preliminary Assessment on Unidentified Aerial Phenomena ^4^ and the most recent (2022) DNI Report on UAP ^13^ list five potential explanatory categories for UAP sightings—airborne clutter, natural atmospheric phenomena, U.S. government or industry developmental programs, foreign adversary systems, and other ^4^.

Early research attempting to explain an increase in sighting reports in Utah’s Uinta Basin uses airborne insect infestation as a correlate. The selected insects showed patterns of “brilliant colored flares or brushes of bluish white light from various external points on their bodies” during electric field stimulation ^17^. The artificially generated electric field was suggested to resemble a weather-related phenomenon called St. Elmo’s Fire, where static electricity causes patterns of visible colored light. Interestingly, this research was refuted soon after publication and described as “somewhat unrealistic,” ^18^ though the authors did respond with a rebuttal ^19,20^.

Other historical research suggests connections between seismic activity and UFO sightings. Persinger and Derr ^21^ recall the tectonic strain hypothesis ^22–24^—“that a substantial portion of UFO phenomena are generated by strain fields; they are evoked by the changing stresses within the earth’s crust” ^25^. Other research suggests that seismic activity connected with solar activity or the use of seismic intensity may be a better predictor than just with seismic activity alone ^26^.

Maybe the most popular natural explanation for UAP sightings is ball lightning, characterized by “a spherical or roughly spherical light-emitting object whose size varies from a few cm to a meter or more, with an average diameter of about 20 cm, and whose colors vary from red to yellow, white, blue, and (rarely) green” ^27^. One of the issues with the ball lightning hypothesis is that it is such a rare, and rarely recorded event, that its existence is not accepted by all researchers. However, relatively recent research has confirmed what is believed to be a ball lightning incident ^28^.

The recent increase in interest in UAP reports has been accompanied by the development of new methods to assess and explain sightings, ^29,30^ including custom built observatories and sensors, as well as mobile apps designed to leverage crowd-sourced information. While these methods bring new sophistication to the analysis of individual events, there remains no information on the general context of sightings, i.e., why sighting reports are more common in certain regions of the country, and less common in others. Rather than attempt to explain what people are seeing in the sky, we explore the combination of visibility and air traffic as it relates to reported sightings, thus providing a first order understanding of why the number of sighting reports varies spatially. Given their relative rarity, it seems unlikely that insects, seismic activity, and/or ball lightning are responsible for the majority of reports, especially those seen in the daytime. Understanding the environmental context of these sightings will make it easier to propose and test new explanations for their occurrence and help to identify any truly anomalous sightings.

## Materials and methods

### Public UAP sighting report data

**Figure 1:**
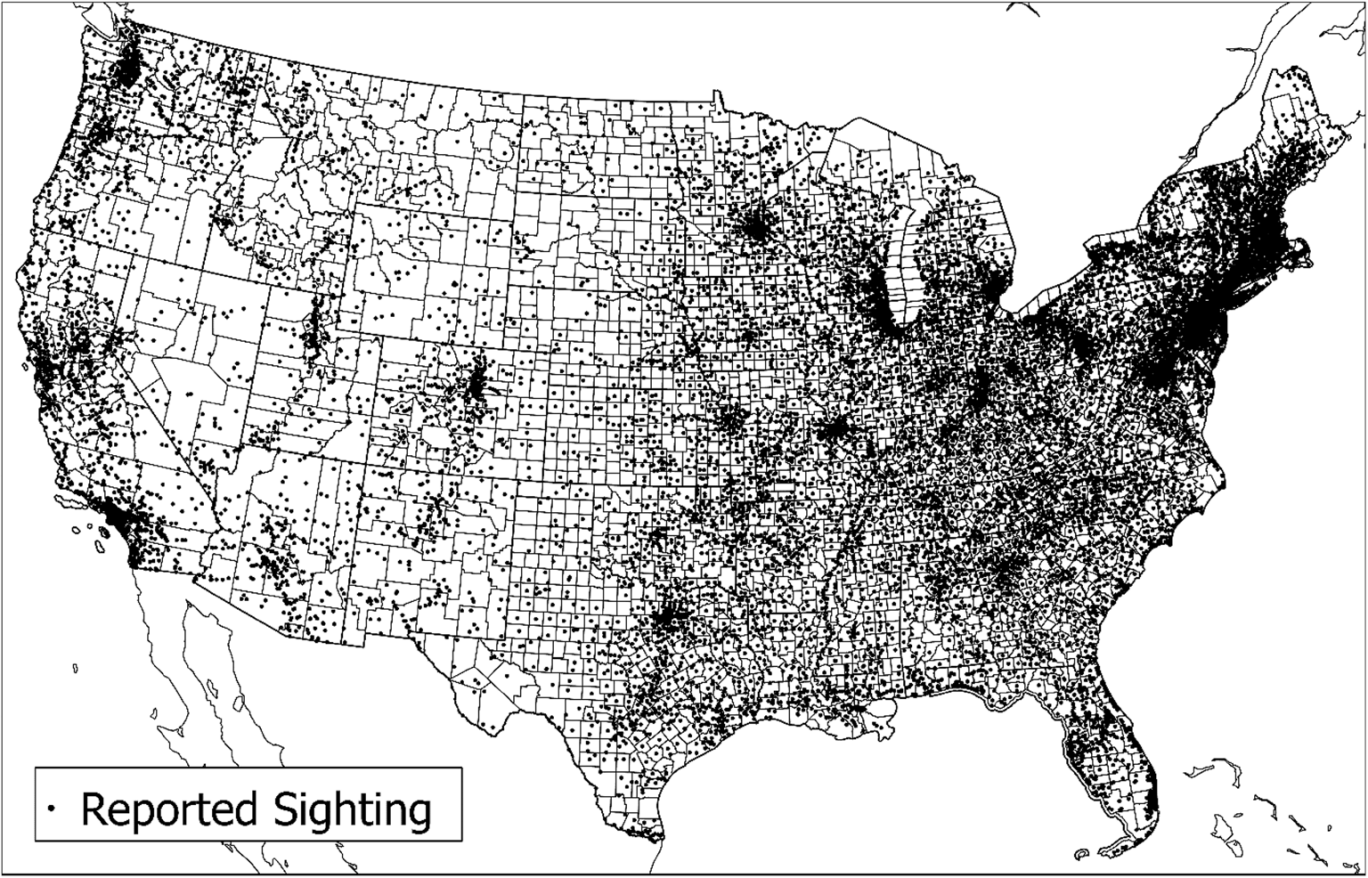
NUFORC Reported Sighting Spatial Distribution for the Conterminous U.S. from 2001 to 2020.

This research uses data from the National UFO Reporting Center (NUFORC) online ^31^. NUFORC was formed in 1974 and “the Center’s primary function over the past four decades has been to receive, record, and to the greatest degree possible, corroborate and document reports from individuals who have been witness to unusual, possibly UFO-related events” ^32^. NUFORC accepts online, phone, and written reports. The data are updated approximately once a month. Our extracted dataset includes 122,983 reported sightings in the United States from June 1930 to June 2022. Fields in the dataset include Date, City, State, Country, Shape, Duration, Summary, Posted Date, and Image. Coordinates at the city level were calculated using Microsoft online services. The resulting spatio-temporal dataset includes 121,949 points (locatable in the United States), which is 99.16% of the total extraction. We focus on the conterminous U.S. from 2001 to 2020 for (1) ease of interpretation and (2) because the tree canopy data (discussed below) are only available for the coastal region of Alaska. This reduces the number of reported sightings to 98,724 (shown in Fig. 1).

**Figure 2:**
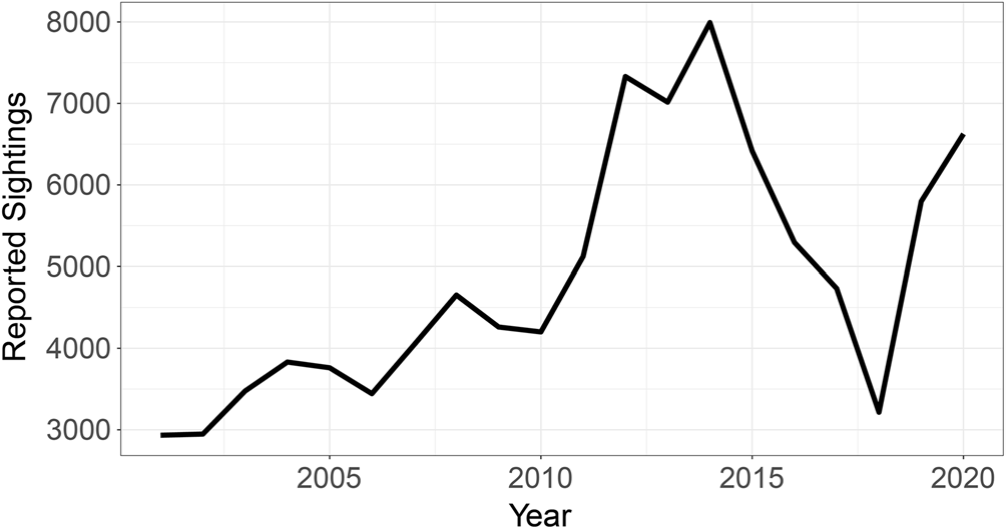
Timeline of NUFORC Reported Sightings from 2001 to 2020.

For analysis, we aggregate to the county level across this time period for spatial continuity. For all spatial studies the Modifiable Areal Unit Problem (MAUP) is always a consideration. While calculating and analyzing sighting reports might be less biased if aggregated to equally sized cells, estimating population within such cells requires a series of assumptions. Also, since these reporting events are relatively rare, counties provide large enough areas for a meaningful aggregation of points. Our temporal range is selected such that entries are assumed to be relatively recent events and not generated from memories decades ago. Internet access to report a sighting would be more possible beginning about 2000 and is likely responsible for the increase in sighting reports over time. Furthermore, from 2000 to 2010 especially, and in rural areas, a potential reporting bias exists due to lessened internet access in those areas. A timeline of reported sightings for the study period is provided in Fig. 2, with a marked peak in reports between 2012 and 2014, followed by a sharp drop between 2015 and 2018.

In the spatial sciences, data like these are typically referred to as Volunteered Geographical Information (VGI). VGI are volunteered either knowingly or unknowingly by individuals, typically with the assistance of location enabled digital tools ^33^. Like with other crowdsourced data, there is little hope for assurance of quality for VGI ^34^. This problem is compounded in this dataset where some may be attempting to disinform. It is clear that these data cannot be verified, and even if interviews with each person were possible, there would be issues determining truth and accuracy, especially for retroactive reporting. However, NUFORC does attempt to limit false reporting. First, they provide information including descriptions, images, and video of Starlink Satellites, which can look unidentified to those that have not seen them before. Second, they provide a description of Venus as a potential for unidentified sighting reports. Third, NUFORC discusses hoax and joke reports, which are said to be ignored and discarded ^35^. Given the size and structure of the data, it is not clear that all hoaxes can be identified, but at least NUFORC is paying attention to hoax cases. We cannot differentiate those sighting reports that have obvious and/or logical explanations, but we note that these still represent an ‘unidentified’ sighting report. However, this is the only dataset of this size and detail that allows for geographic research. Furthermore, it is impossible to discredit over 120,000 cases.

### Explanatory variables

We use 3 explanatory datasets to represent physical and built environment attributes that would restrict the view of the sky: light pollution, cloud cover, and tree canopy, and 2 datasets that represent airborne activity that might be mistaken for UAPs. All data preparation and calculations are made using Microsoft Excel and ESRI ArcGIS Pro software. All covariates were z-score transformed prior to modeling.

**Light pollution** The data source for light pollution is the New World Atlas of Artificial Sky Brightness ^36,37^. This raster data set is available in geotiff format with 30 arcsecond or 1 km per pixel resolution and covers the entire world. Values represent simulated zenith radiance in [mcd/m^2^]. The data for the U.S. were extracted and the mean value for light pollution was calculated for each U.S. County.

**Cloud cover** Cloud cover data are sourced from the EarthEnv Project ^38^. These data are compiled using 15 years (2000–2014) of twice-daily remotely sensed observations from the Moderate Resolution Imaging Spectroradiometer (MODIS) sensor. They are provided in geotiff format at 1 km resolution for the entire world. The cloud cover values were averaged for each U.S. County.

**Tree canopy** The tree canopy data are from the Multi-Resolution Land Characteristics Consortium and created by the United States Forest Service (USFS) using Landsat imagery and “other available ground and ancillary information” ^39^. Tree canopy estimates cannot be calculated by spectral signature alone. Here they are generated using random forest models that are trained on manually classified Digital Orthophoto Quarter Quadrangles (DOQQ) as response variables ^40,41^. This helps estimate the difference between tree canopy and other vegetative land cover. The resulting data values represent 2016 canopy cover at 30 m resolution and are available for the continental U.S., coastal Alaska and Hawaii. Because of the size of the file and the resolution of other datasets in the model, the image was upsampled to 1 km resolution. The tree canopy values were then averaged for each U.S. County.

**Airports** These data are provided by ESRI’s, ArcGIS Online service accessible through the ArcGIS Pro software. They include categories for airports, heliports, seaplane bases, ultralights, gliderports, balloonports, and other in the U.S. There are 19,850 entries in this dataset represented as points. The data are standardized as the number of airports per sq. km.

**Military installations** Military installation data are sourced from U.S. Census TIGER/Line shapefiles and downloaded from data.gov ^42^. The U.S. Census created this dataset in collaboration with the U.S. Department of Defense and the U.S. Department of Homeland Security. The data delineate the boundaries of military installations. For this research, those boundaries were overlaid onto U.S. counties, where the area of military installation of each county is calculated.

### Models

We first explore the NUFORC dataset using the Getis-Ord (Gi*) index based on the number of sighting reports per 10,000 people per county. This identifies significant clusters of low values (cold spots) and high values (hot spots), by comparing the aggregate number of standardized reports in a set of neighboring counties to the full distribution. The neighboring counties are selected as k-nearest neighbors (k-NN) with the K parameter set to 8. Rather than setting a fixed distance parameter or contiguity requirements, k-NN ensures that each county considers the same number of neighbors. The population standardization of the sighting report variable should help correct for regions with larger counties, such as the West, which generally cover larger areas ^43,44^.

To model potential for seeing UAPs we use Bayesian small area estimation, based on the relative rate of sighting reports in the population of a location. Small area models incorporate a spatial autoregressive term to limit the influence of extreme values, which can result from small population sizes. Here, the count of reported sightings ${y}_{i}$ for county *i* is assumed to follow a Poisson distribution:

$$ y_{i} \sim Pois\left( {\theta_{i} E_{i} } \right) $$

where $E_{i}$ is the expected number of reports for county *i* and $\theta_{i}$ is the relative rate. To get the expected value, first we estimate the per capita rate of reports for the conterminous U.S. as the total number of reports divided by the total population. The expected value for any county is obtained by multiplying this value by the population of that county. Where $\theta_{i} > 1$, the number of reports is greater than would be expected based on population alone. A recent analysis of the NUFORC dataset suggests that the number of reports may also be linked to county area ^45^. However, given that the distribution of the population in a given area may be highly variable, it is unclear how to use this in the expected rate calculation. We therefore assume that the expected rate of reports is based simply on the capacity of a county to produce reports. Finally, the relative rates are modeled using K covariates as follows:

$$ \log \theta_{i} = \mathop \sum \limits_{k = 1}^{K} \beta_{k} x_{ik} + \in_{i} $$

where $\mathop \sum \limits_{k = 1}^{K} \beta_{k} X_{ik}$ represents the set of z-score transformed covariates representing visibility and air traffic described above with associated coefficients. Finally, the model error ($\in$) is decomposed into a spatial autoregressive effect and non-spatial random noise. Our model assumes that the individual reports are independent. While this is unlikely to be true of the events that caused the sightings, as these may be reported by multiple individuals, we assume that the reports are independently provided.

Model parameters and coefficients are estimated using Integrated Nested Laplacian Approximation (INLA) ^46^. INLA was chosen over MCMC approaches due to its computational efficiency with large spatially structured models. Model results are reported as the mean of the posterior probability distribution for each coefficient (Table 1). Variance Inflation Factors (VIFs), which signal potential multicollinearity within a model, for all variables in the model are well under 2. VIF values are traditionally accepted if they are under 5. Bayesian posterior estimates can be used to test specific hypotheses ^47^. Here, we test the hypotheses that the relationship between each covariate and the rate of sighting reports is positive (i.e., > 1) or negative (< 1). Support for a given hypothesis is based on the posterior probability distribution of model coefficients and is described as the credibility of that hypothesis. For example, if 95% of the posterior distribution of a coefficient is above one, this indicates a positive relationship between that covariate and the rate of sighting reports and would be assigned a credibility of 95% of a positive relationship. If the posterior distribution is equally split into negative and positive estimates, this would be assigned a credibility of approximately 50% for either hypothesis. As the model is based on log-transformed relative rates, the posterior estimates of coefficients have been exponentiated to help in interpretation. Coefficients are reported as the mean of the posterior distribution plus the 95% credibility interval (Table 1). A map of the spatial error term (u) is included in supplementary information.

**Table 1:** Results from Bayesian small area model.

Variables	Exponentiated Results	Positive coefficient credibility (%)	Negative coefficient credibility (%)	Relationship
(Intercept)	0.862 (0.848, 0.877)			
Mean Canopy	0.961 (0.915, 1.01)	6	94	More Canopy = Fewer Reports
Mean Cloud Cover	0.998 (0.929, 1.072)	48	52	No Credible Relationship
Mean Light Pollution	0.923 (0.899, 0.947)	0	100	More Light = Fewer Reports
Percent Military Area	1.013 (0.994, 1.033)	92	8	More Military = More Reports
Air Traffic/Sq. Km	1.099 (1.068, 1.131)	100	0	More Air Traffic = More Reports

From left to right: variable name; mean posterior distribution (95% credible range); credibility of positive relationship with sighting reports; credibility of negative relationship with sighting reports; brief description of result.

## Results

**Figure 3:**
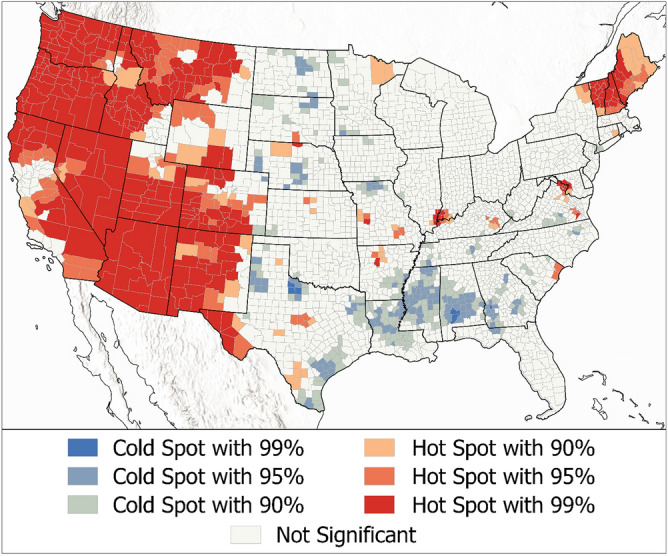
Hotspot Analysis (Getis-Ord Gi*) of Reported Sightings from 2001 to 2020.

The results from a hotspot analysis (Fig. 3) show a strong trend with many more population standardized sightings (i.e., county reports per 10,000 people) reported in the Western U.S. and in the very Northeast, along with some isolated areas including the tri-state border region of Illinois, Indiana, and Kentucky, surrounding Evansville, Indiana, and the area surrounding Washington D.C. Clusters of low sighting reports are found through the central plains and in the southeast.

Table 1 provides the results of the model, based on the posterior probability distribution of each coefficient. With the exception of the intercept, all model coefficients describe the rate of change of the relative rate of sighting reports for a one standard deviation increase in that coefficient. Values above 1 indicate a positive relationship (i.e., increasing reports); values below 1 indicate a negative relationship (decreasing reports). For example, the coefficient for Mean Light Pollution is 0.923, indicating that a one standard deviation increase in light pollution will result in a 7.7% decrease in sighting reports.

All results except for cloud cover support the general hypothesis that people will see things when they have the opportunity to. Cloud cover has a non-credible relationship with sighting reports, with no support of either a negative or positive relationship.

## Discussion and conclusions

We recall here our initial research questions: (1) What is the viability of publicly offered data on reported UAP sightings? (2) Are there credible spatial patterns to these sighting reports? and (3) If so, can these patterns be explained by physical and/or built environment factors? For question 1, the publicly available data from NUFORC online are useable data; however, they require substantial processing for spatial analysis. These data could be used for finer resolution (city level) research, rather than county level used here.

The main concern of these findings is, are these volunteered data valid? The short answer is that it is likely that some are and some aren’t. However, we suggest that if the data were entirely invalid (assuming homogeneous psychological and sociological distribution of submissions), the sighting reports would exhibit little to no spatial pattern and are unlikely to follow a pattern that can be explained by first-order visibility indicators. Another data question is, are there any temporal and/or geographic errors? Likely, because some entries into this dataset are reported retrospectively, not always in the first person. We attempt to limit this by using data from 2001-present, but that does not completely resolve the issue. Geographic errors were limited by upscaling the data to the county level. A final issue we consider is that these reported cases require knowledge of NUFORC and access to communications. The authors found the website and organization while searching for data. Some may find the website while searching for an organization to report to. Still, there is likely bias in who has knowledge of this resource since it is not widely advertised. In all, we posit that this dataset has value in understanding these sighting reports; that either this indicates people are seeing things they can’t explain (or that they don’t want to explain with more logical explanations), or this indicates where people are thinking more about UAPs. Both are important and have physical/social implications.

For questions 2 and 3, there are credibly identifiable patterns to these sighting reports, and these patterns relate to environmental characteristics. The explanatory variables are intended to represent both (1) the opportunity to see something and (2) the potential for something human constructed to be in the field of view. We have not considered satellites or drones, which are likely important factors, nor the fact that airplanes (and helicopters, etc.) do not only fly around their takeoff and landing locations. However, around the locations we use, aircraft are likely to be closer to the ground, more visible, and more frequently present. Using the military installation data, we hope to capture, not only aircraft, but also nighttime training activities that might use, for example, tracer rounds, drones, and other forms of illumination in relatively desolate areas.

If we assume that most sighting reports here are representative of true sightings that people determined to be unidentified, then our results have interesting implications. Our model shows that the majority of standardized sighting reports are in the western parts of the U.S. and in the very northeast. We hypothesize that the higher rate of western sightings could be due to (1) the physical geography of the West (i.e., the lack of vegetative canopies and wide-open spaces), (2) cultures of outdoor activity (e.g., recreation and other activities enjoyed in more temperate weather throughout the year), and (3) cultures of paranormal ideation (e.g., impacts of Area 51, Roswell, New Mexico). There are also some isolated counties throughout the rest of the country that warrant further investigation to identify what properties may generate relatively more UAP attention. In these results, however, cloud cover is not credible, possibly related to higher rates of sighting reports in both the coastal regions of the Pacific Northwest (relatively clouded) and desert regions of the Mountain West (relatively clear). We initially expected cloud cover to be credibly related to reports, as clouds can cause light to scatter and by doing so, obscure reflective or illuminated things that are moving within or above them and create patterns that some might consider unexplained. However, that was not the case. All other variable relationships are as expected and align with our initial hypotheses, that people report more sightings where they have a better view of the sky. The question now is why? This research begins to answer this question by considering how much human made airborne activity is occurring. The highly credible relationships with air traffic and with military activity suggest that people are seeing, but not recognizing, things that are human made. As an example, a hot air balloon seen from a far enough distance can look unexplainable, especially if it is seen by someone who has not seen one before. Drones, which we did not test specifically for, can seem to fly erratically in areas where people aren’t used to seeing things moving in the sky. It is unlikely that events, such as ball lightning, seismic based lights, insects, or other natural occurrences are responsible for more than a small portion of these reports, as they are rare events themselves.

While these results provide an initial assessment of factors linked to the reported sightings of unidentified or unexplained phenomenon, they also generate further questions. We find credible relationships and spatial patterns that require further investigation. Why, for example, are the rates of sighting reports low in California, when they are high in many of the surrounding states? Why do the rates of reports fluctuate across time? Our future research will include temporal considerations (e.g., variation over time) to hopefully address some of these questions. We further note that our covariates represent average conditions, and while these clearly explain much of the first-order pattern in sighting reports, additional factors may be identified by exploring the remaining pattern in the spatial errors (SI Fig. 1) or by considering changes over time or individual events.

Some patterns in the reported sightings might be explained by sociocultural factors. For example, are there spikes of reports after Hollywood attention is given to movies or TV shows on aliens? Are some cultures more likely to see UAPs, because of their belief systems? Have some U.S. regions/places been given more attention to historical UAP sighting reports? There is no question that geography and “place” influence people’s belief systems and behavior. In some places, the expectation of what you are supposed to see may influence what you actually see. In a process termed *motivated perception*, people may bias their perceptions to arrive at expected conclusions that meet their goals or offer rewards ^48,49^. If your goal is to see a UAP, you may very well see one given the opportunity. However, it is important to point out that there are many sighting experiences which people are reluctant to report. There are many who fear stigmatization and attacks from the public, and others who previously had no belief in UAPs, but had an experience that convinced them of the opposite.

We approach this problem with caution, because of both the complexity of the topic and the sensitivity of available data. The U.S. Government position is that “UAP clearly pose a safety of flight issue and may pose a challenge to U.S. national security” ^4^. For national security issues, uncertainties and unknowns are never good, and it is the job of intelligence efforts to minimize the unknowns. Regardless of what people are seeing, and whether they are military pilots, civilian pilots, or general bystanders, there is a potential threat. That threat grows as our uncertainties grow. Although based on a noisy, crowd sourced dataset, our results can provide a context for how sighting reports of unidentified objects vary in space, the factors linked to these, and may offer a step towards understanding these threats.

This problem is relevant on many fronts, including anthropological and sociological (i.e., understanding the human/social experience). The stigma given to this area of research, if it is explored scientifically, should be over. We make no hypotheses about what people are seeing, only that they will see more when and where they have opportunity to. The question remains, however, as to what these sighting reports are of. Further examination of regions where the model performs poorly, temporal trends, and reported details of each reported sighting may help further elucidate this.

### Supplementary Information

Supplementary Figure 1.

## Data Availability

The data that support the findings of this study are available online from the National UFO Reporting Center (NUFORC) at https://nuforc.org/; however, these data are not geocoded. Geocoded data are available from the authors upon reasonable request.
