# Supplementary figures and images for: An environmental analysis of public UAP sightings and sky view potential
Source: Sci Rep. 2023 Dec 14;13:22213. doi: 10.1038/s41598-023-49527-x (PMC10721628; doi:10.1038/s41598-023-49527-x)

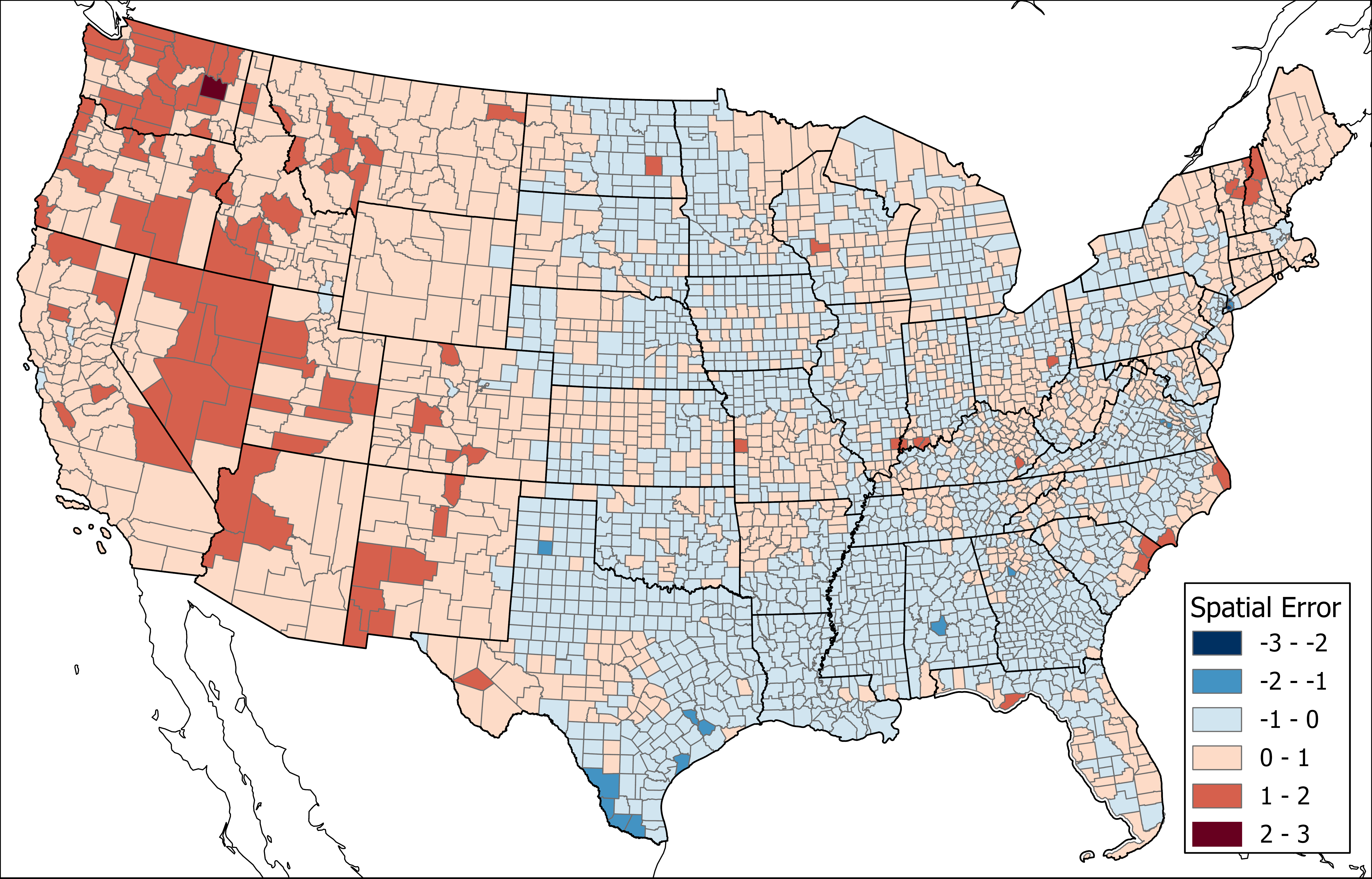


**Fig. 1.** Posterior mean of the spatial error from the BYM2 model. Units are the log of relative rate.

Supplement: Supplementary file 1 — Supplementary Figure 1. [file 41598_2023_49527_MOESM1_ESM.docx]
